# Navigating the shadow of fear: a qualitative study unveiling the lived experience of fear of cancer recurrence among Chinese gastric cancer survivors undergoing chemotherapy

**DOI:** 10.3389/fmed.2025.1704087

**Published:** 2025-12-02

**Authors:** Haiyan Zhao, Ye Zhou, Yuzhu Hou, Yun Liu

**Affiliations:** 1 Jingjiang People's Hospital Affiliated to Yangzhou University, Taizhou, Jiangsu, China; 2 Department of Nursing Science, Faculty of Medicine, Universiti Malaya, Kuala Lumpur, Federal Territory of Kuala Lumpur, Malaysia; 3 International Journal of Nursing Sciences, Chinese Nursing Journals Publishing House Co., Ltd., Beijing, China

**Keywords:** cancer survivors, social isolation, descriptive qualitative research, thematic analysis, fear of cancer recurrence

## Abstract

**Purpose:**

This study aimed to gain an in-depth understanding of the psychological experience associated with the fear of cancer recurrence (FCR) among survivors of gastric cancer. Additionally, it sought to explore the triggering factors that trigger this fear and the coping strategies employed by individuals facing this challenge.

**Methods:**

The Purposive sampling method was used to select study participants. A total of 20 survivors with gastric cancer who had undergone chemotherapy. Participants were recruited from a tertiary hospital in Jingjiang City, Jiangsu Province, and data collection took place from November 2023 to January 2025. Semi-structured, in-depth interviews were conducted during the chemotherapy sessions, with each interview lasting 30 to 45 min. The interview data were analyzed using the thematic analysis method.

**Results:**

The psychological experience of FCR among survivors of gastric cancer was categorized into three main themes: (a) Varied manifestations of the FCR: including self-perceptions, impact on family members, and the surrounding atmosphere; (b) Factors triggering the FCR: such as symptoms, treatment, environment, and future plans; (c) Coping strategies for the FCR: involving both positive and negative coping mechanisms.

**Conclusion:**

Recognizing the pervasive nature of the FCR, it is essential to implement comprehensive interventions that encompass personal, family, and societal dimensions. Such interventions can help alleviate the sense of disease uncertainty experienced by survivors of gastric cancer. By adopting a holistic approach, a support model involving the hospital, family, and society can be developed to provide robust assistance for individuals navigating the challenges of cancer.

## 1 Introduction

Gastric cancer is the second most prevalent form of cancer in China, with a total of 679,000 cases and 498,000 deaths, second only to lung cancer, which accounts for 733,000 deaths (1). In China, gastric cancer causes 9.825 million disability-adjusted life years, representing 44.21% of the global burden (2). Survivors with cancer are defined as individuals who have been diagnosed with cancer and are still alive, encompassing their entire lives following diagnosis (3). The survival period for survivors of gastric cancer has been significantly extended in recent years due to advancements in medical technology, leading to a growing population of gastric cancer survivors. Fear of cancer recurrence (FCR) refers to the psychological fear experienced by cancer survivors of cancer regarding the recurrence, progression, or metastasis of cancer in the primary or other parts of the body (4). A survey reported that among gastric cancer patients undergoing chemotherapy, 69.9% of patients had low FCR, whereas 30.1% had high FCR (5). Research has shown that receiving chemotherapy is positively associated with the level of fear of cancer recurrence (FCR) in cancer patients (6, 7). FCR is particularly pronounced during the chemotherapy phase, which often extends over a prolonged period and imposes considerable physical and psychological burdens on patients. Consequently, FCR tends to peak during this treatment period, negatively affecting treatment adherence, recovery, and daily functioning (8, 9). This fear has far-reaching consequences, impacting not only the physical and mental well-being of survivors of gastric cancer but also giving rise to familial and social issues. Currently, clinical staff commonly employ diverse quantitative evaluation tools to assess the presence of fear of progression among survivors (10, 11). While these tools are user-friendly, they may not promptly detect cognitive abnormalities in survivors, and they may struggle to unveil the specific manifestations and underlying causes of their fear. Most studies on the FCR have predominantly focused on survivors of breast cancer (12, 13), with a notable absence of studies specifically examining the FCR among survivors of gastric cancer. Several studies (14–16) have shown that survivors with gastric cancer exhibit higher levels of FCR, compared to survivors with breast cancer (17, 18) and early-stage prostate cancer (19). This discrepancy may be attributed to the prolonged treatment cycle and higher recurrence rates commonly associated with advanced gastric cancer (16). Furthermore, survivors of gastric cancer often experience adverse gastrointestinal reactions, including nausea, vomiting, dry mouth, constipation, and pain, which significantly amplify their psychological burden and intensify their fear of disease recurrence and progression (16). Additionally, research conducted by Zhang and Ding et al. (6, 19) revealed that survivors’ FCR tends to be higher during chemotherapy compared to the follow-up period. Therefore, the present study conducted qualitative interviews with survivors, specifically during chemotherapy. The primary objective of this study was to gain an in-depth understanding of survivors’ psychological experiences regarding the FCR. Additionally, the study aimed to explore the factors that trigger this fear and investigate the coping strategies employed by individuals facing this formidable challenge.

## 2 Materials and methods

### 2.1 Ethical approval

This study was approved by the Ethics Committee of Jingjiang People’s Hospital (KY 2022–167-01) and adhered to the principles of the Declaration of Helsinki. All participants were informed of the purpose and procedures of the study, and written informed consent was obtained from them before the data collection procedure.

### 2.2 Participants and recruitment

This study utilized purposive sampling to select participants based on diverse characteristics, including gender, age, residential environment, medical burden, work status, previous gastric surgery, and cancer diagnosis time. Interviews were conducted with hospitalized survivors of gastric cancer who were hospitalized at the oncology department of a tertiary hospital in Jingjiang, Jiangsu Province, China, from November 2023 to January 2025.

#### 2.2.1 Inclusion criteria

- The survivor’s disease meets the diagnostic criteria for “Guidelines for Diagnosis and Treatment of Gastric Cancer” (20). The preoperative gastroscopy and pathological diagnosis indicate advanced gastric cancer.
- Undergoing chemotherapy.
- age≥ 18 years; able to communicate effectively; the main caregiver is their spouse.
- Clear consciousness; no understanding barriers.
- Provided written informed consent.

#### 2.2.2 Exclusion criteria

- Another diagnosis of gastric cancer or another type of cancer prior to the study.
- Presented with severe complications such as gastrointestinal obstruction or perforation.
- Has received or is currently receiving psychotherapy from a psychiatrist or psychologist.
- Cognitive impairment or mental impairment.
- Severe visual, hearing, and speech impairments.

The sample size for this study was determined based on achieving data saturation, which was reached after collecting interview data from 15 participants, and no new information or themes emerged. To further confirm data saturation, an additional five interviews were conducted, resulting in a total of 20 participants included in the analysis. When selecting participants, consideration was given to their different living environments, cancer treatment burden, and chemotherapy cycles were considered whenever possible. The characteristics of the survivors are presented in Table 1, which includes 15 female and five male survivors. The mean age of the participants was 62 years, ranging from 45 to 73 years. Furthermore, a notable proportion of the survivors were not employed (*n* = 17, 85%) and had undergone gastric surgery (*n* = 14, 70%).

**Table 1:** Participant characteristics (*n* = 20).

Demographic	*n* (%)
Gender	
Female	15(75.0)
Male	5(25.0)
Age	
41–50 years old	1(5.0)
51–60 years old	6(30.0)
61–70 years old	6(30.0)
>70 years old	7(35.0)
Residential environment	
City	9(45.0)
Current chemotherapy cycle	
>2个周期	11(55.0)
≤2个周期	9(45.0)
County/Town	6(30.0)
Rural	5(25.0)
Cancer treatment burden	
Completely free	3(15.0)
Basic burden	9(45.0)
Some burden	5(25.0)
Heavy burden	3(15.0)
Work status	
Working	3(15.0)
Not working	17(85.0)
Previous gastric surgery	
No	6(30.0)
Yes	14(70.0)
Cancer diagnosis time	
Less than 1 month	1(5.0)
1–2 months	5(25.0)
2–3 months	7(35.0)
More than 3 months	7(35.0)

### 2.3 Data collection

A preliminary interview outline was developed based on an extensive literature review. To ensure its validity and appropriateness, five experts from the fields of oncology, psychology, and gastrointestinal medicine were consulted. Additionally, three survivors with gastric cancer were selected for pre-interviews, although their data were not included in the analysis. A refined interview guide was formulated by engaging with the survivors and seeking their perspectives, as well as receiving valuable guidance and suggestions from the expert panel.

The interviewer initiated the interviews by establishing a rapport with the participants through the question, “Can you share your experience with the disease?” The main interview questions included:

- How has the disease affected your life?
- Are you worried or afraid of cancer progression? Please provide specific examples or situations.
- Under what circumstances does this fear or anxiety occur?
- Does this fear or anxiety affect your daily life, family, or work? How do you cope with it?
- What other emotions do you experience during treatment? Are there any special thoughts or feelings you would like to express?

The data was collected through semi-structured, in-depth interviews. To minimize the potential influence of medication on survivors’ psychological state, the time of interviews was conducted after the patients were hospitalized but before the chemotherapy drug infusion. Each interview lasted between 30 and 45 min to ensure the participants ‘convenience and comfort. The interviews were conducted in a quiet, comfortable room with the door closed to ensure privacy and minimize interruptions from hospital staff, thereby maintaining confidentiality for the survivors. The atmosphere was kept open and relaxed to help create a calm environment for the interviewees. The two interviewers were not part of the patients’ direct caregiving team but were research nurses from the oncology department. Due to their work in oncology, they were well-acquainted with the patients’ conditions and had established rapport with them, which facilitated trust and open communication during the interviews. Before beginning with each interview, the researcher provided a clear introduction to the background and significance of the study. Written informed consent was obtained from each participant. Throughout the interview process, the researcher took detailed notes and recorded the entire conversation with the explicit consent of the survivors.

### 2.4 Data analysis

After each interview, the researcher transcribed the recorded conversation into text and supplemented it with on-site notes within 24 h. Data sorting and analysis were conducted simultaneously using the thematic analysis method for qualitative analysis. The specific steps involved in data analysis were as follows.

The specific data analysis steps were as follows:

The first and fourth authors thoroughly read and reviewed the interview data repeatedly until they gained a comprehensive understanding of the overall data. Each line of the data was carefully analyzed, and statements of significant importance were identified. Open coding was conducted to categorize and label the data based on emerging themes and patterns. Any differences in coding that emerged were resolved through discussion and consensus among the researchers. Once consensus was reached on the coding process, the two authors compared and classified similar or related codes, gradually forming subthemes and themes.

The authors systematically defined and documented the codes, subthemes, and themes. The themes underwent a thorough review, improvement, and naming process until the final coding hierarchy was established (Table 2). Representative examples were extracted from the data as evidence to support the identified themes. Strict measures were taken to maintain the anonymity and confidentiality of the participants. All participants were assigned anonymous identifiers (N1 to N20) to safeguard their privacy throughout the interview process.

**Table 2:** Coding hierarchy.

Theme	Subtheme	Major codes
Varied manifestations of the fear of cancer recurrence: from themself, family members and the atmosphere	Persistent fear and pessimistic attitude	Mild fear
		Intense fear
		fear of cancer recurrence that persists
	Suspicion and vigilance regarding cancer progression	Frequently doubting disease progression
		Maintaining a high level of vigilance
		Fear of hearing medical **examination** results
	Fear related to family members	fear of cancer recurrence in survivors’ family members
		Fear of causing trouble and psychological distress
		Fear of cancer gene inheritance
Factors Trigger the fear of cancer recurrence: symptoms, treatment, environment and future plans	Perception of one’s symptoms and defects	Alert to signs of cancer progression in the body
		React excessively
	Treatment, examination, and communication with healthcare providers	Go for hospital re-examination
		Increase other **examination**s
		Healthcare providers’ response is not timely
	Surrounding environment and the lack of understanding	Talking about cancer by others
		Being misunderstood by others
		Needing to rely on others
		Others’ distancing and cancer-related shame
	The stress arising from making future plans	Holding a pessimistic attitude towards future plans
		Economic pressure is more likely to trigger fear and anxiety
Coping strategies Coping Strategies for the fear of cancer recurrence: Positive coping and negative coping coexist	Avoidance of fear, autonomously	Avoiding contact with anything related to cancer
		Choosing to reduce medical examinations independently
	Self-endurance	Enduring silently by oneself
		Decreasing the psychological burden on others
	Accepting reality	Accepting the fact of cancer
		Negative Coping strategies
		Distracting oneself
		Adjusting one’s mindset
	Healthy belief and Lifestyle	Positive cognitive behaviors
		Cultivating healthy lifestyle habits
		Especially paying attention to diet
	Seeking help and support from family members	Family members assisting survivors in cultivating healthy lifestyle habits
		Caregivers changing unhealthy habits
		Family members providing psychological comfort to survivors
		Inter-generational communication and support
	Seeking professional and social support	Doctors providing disease information
		Nurses providing care and emotional support
		Peer support

### 2.5 Quality control

Both interviewers in this study are experienced oncology nurses and hold qualifications as psychological counselors. They have extensive expertise in conducting semi-structured interviews and have received comprehensive training in qualitative research methods. The participants voluntarily chose to participate in this study, and prior to the interviews, the researchers established a strong foundation of trust and rapport with them. Throughout the interviews, the researchers attentively observed and recorded nonverbal behaviors, including facial expressions, body movements, and speech pauses displayed by the participants. After each interview, the researchers engaged in a reflective process, documenting their thoughts and insights in a reflection diary and memo.

To enhance the rigor of the study, the researchers employed a triangulation method. This involved having two researchers conduct interviews with participants at different times, locations, and with varying personal characteristics. This approach ensured the reliability and validity of the research findings by corroborating and strengthening the consistency and trustworthiness of the results through multiple perspectives and sources of data.

During the data analysis, the researchers adopted a neutral stance, suspending their personal views and avoiding any interference from their perspectives and previous experiences. This approach aimed to minimize biases that could arise from the researchers’ values and emotional involvement in the analysis process. To further ensure the accuracy and authenticity of the data, participant checking will be conducted. The researchers will provide the transcribed data to the participants for confirmation, allowing them to review and validate the accuracy and authenticity of their contributions. Overall, the researchers employed various strategies and methodological precautions to ensure the rigor, credibility, and trustworthiness of the study.

## 3 Results

### 3.1 Varied manifestations of the FCR: from themself, family members, and atmosphere

#### 3.1.1 Persistent fear and pessimistic attitude

Faced with the possibility of cancer progression, survivors exhibited a wide range of fear responses. Some survivors had mild fear, but most experienced strong fear, manifested as loss of appetite, sleep disorder, irritability, indifference, loss of hope, and a pessimistic outlook.

*"I am not very worried about progression because my prognosis is good" (P1). "I was diagnosed early, so my prognosis is still good, and I'm not very afraid" (P15).*

*"I am afraid of the pain in my stomach (after progression), and worry that I won't be able to eat. I have lost weight now"(P4). “I usually worry at night and can only take tranquilizers to help me fall asleep"(P20). "Because my body's resistance is weak, I feel scared with any pressure around me. Now I get angry easily and want to lose my temper for no reason"(P9). "I can't play cards anymore (silent), and I'm not interested in anything" (P17).*

*"I have given all my money to my son and grandson"(P20). "I feel like I only have a few years left to live. I have to take medicine for the rest of my life. It's better to die" (P13).*

Some survivors indicate that their FCR persists because of unfamiliarity with the disease itself, and the fear tends to recur:

*"I don't know what's going on. Many times, I worry because I don't know what's going on, so I worry all the time" (P14).*

#### 3.1.2 Suspicion and vigilance regarding cancer progression

1. **Disruption to daily life**

Survivors often grappled with a sense of uncertainty regarding the progression of the disease, frequently doubting its recurrence and advancement. The enduring sense of uncertainty made survivors highly vigilant about the FCR:

*"I have become overly sensitive. I'm terrified of any small problem on my body, afraid that the disease will become more severe, and I keep thinking about it" (P2). "I maintain a high level of vigilance in life, paying attention to all aspects, and it is easy to turn small things into disasters. I feel very tired" (P7).*

The tense emotions of survivors have even affected their normal lives. Fear of the body cannot keep up with activity as usual.

*"I used to dance in a square, but now I cannot anymore because I'm afraid my body can't keep up" (P5).*

2. **Doubting treatment efficacy**

Survivors even harbored skepticism about the treatment, coupled with a fear of receiving medical examination results:

*"I'm worried that when I undergo chemotherapy, some of my healthy cells will be killed, leading to more severe illness progression. I'm afraid to hear any medical examination results"(P5). “Why do I need to use so many drugs for chemotherapy? I'm very worried every time I use a lot of drugs. I'm afraid of deterioration. Sometimes it is useless to accept chemotherapy, which leads to a decrease in white blood cells and platelets, making me even more worried about my body" (P14).*

#### 3.1.3 Fear related to family members

Family members had a certain degree of fear regarding the progression of cancer, fearing that the survivor’s illness would worsen:

*"I didn't hear my wife calling me to get up in the morning, and she was very nervous, afraid that I had passed away" (P4).*

Some survivors worried that the cancer would progress again, bringing financial pressure to the family. They felt they were unable to contribute to the family, especially afraid of bringing pressure to the children, and causing trouble and inflicting psychological pain on others. The survivors also feared the hereditary nature of their cancer genes:

*"My eldest daughter's husband is also not in good health. His kidney function is not good, and he cannot work. So, I'm very worried and afraid that I won't be able to do it myself"(P11). "I'm not afraid of death itself. I'm afraid of leaving my family (crying); I'm particularly worried about my children's future lives"(P2).*

*"Cancer has a family history, and I'm always worried that cancer will appear in my child's body and affect their life in the future"(P1).*

### 3.2 Factors triggering the FCR: symptoms, treatment, environment, and future plans

#### 3.2.1 Perception of one’s symptoms and defects

Survivors remained constantly attentive to their symptoms and surgical wounds, often prone to overreacting in their response. Gastrointestinal symptoms, including nausea, vomiting, loss of appetite, and changes in stool color, commonly acted as triggers of the FCR:

*"Whenever I feel uncomfortable in my stomach and have nausea and vomiting, I become very vigilant, wondering if the disease has become more serious.....My appetite has been poor since the surgery. I am still very scared"(P4). “Especially when my stool changes color, I worry a lot"(P14).*

Pain serves as another trigger for the survivors, further intensifying their concerns and fears:

*"Whenever I feel pain in my anus, I am very scared, wondering if the disease will progress"(P15).*

Previous surgical wounds also acted as triggers for them, evoking heightened attention and anxiety.

*"Every time I see the protruding scar on my stomach, I become very scared"(P18). “The surgical incision on my stomach hurts, and I feel pain when I eat hard things, which makes me feel even more scared"(P9).*

Concurrent diseases that altered or complicated their condition, such as new or coexisting diseases, or other changes in the body, also served as triggers to exacerbate their fear:

*"I also have heart disease. Every time I feel uncomfortable, I worry that it will affect the progression of cancer"(P4). "My blood sugar is high, and if it is not well controlled, I feel that cancer will become more serious"(P11). "Now I have intestinal obstruction, which makes me very worried" (P17). "I have bleeding and bruises on my body, and my blood pressure is still very low"(P12).*

*“There is a tube in my hand, and I feel a little itchy. I feel scared"(P8). “I fell at home because of dizziness, so I am even more scared"(P17).*

#### 3.2.2 Treatment, examination, and communication with healthcare providers

Survivors were afraid to return to the hospital for disease re-examination, fearing that the results might reveal unfavorable outcomes:

*"Every time I think about having to stay in the hospital for chemotherapy, my heart drops (describing extreme fear)” (P16)."I get annoyed every time I come to the hospital for examination. I feel like exploding with anger, and I'm very sad too"(P10). “I'm very worried before coming to the hospital, afraid that the doctor will tell me the bad news. Every time I have an examination, I worry about whether something bad has happened" (P3). "I don't want to come to the hospital for treatment, and I don't want to have re-examination either. I'm afraid of the doctor's request for re-examination. I'm scared and don't want to come" (P11).*

Additional medical examinations can easily trigger fear among the survivors due to their lack of understanding of the measurement methods and purposes involved:

*"I'm afraid that other medical examinations may affect my disease. I don't understand things like interventional therapy, and I'm very worried about the risks and afraid that they will cause cancer to progress" (P14). “Whenever I have an enhanced CT scan, I'm afraid that my condition will change" (P20).*

The survivors’ uncertainty can increase if healthcare providers do not respond promptly or provide vague answers to their inquiries:

*“The doctor's words are uncertain, and I feel that he is hiding something. I always feel that cancer may progress elsewhere" (P2).*

The imposition of excessive restrictions on the survivors’ daily lives and diet by healthcare providers can also trigger the FCR:

*“There are many requirements for daily life, especially in terms of diet. I can't eat whatever I want, and I'm afraid that my diet is not right, which may cause progression" (P11).*

#### 3.2.3 Surrounding environment and the lack of understanding

The survivors’ FCR was often influenced by their surrounding environment. When they heard others discussing cancer-related topics, or if someone in their vicinity experienced a worsening condition, it could further intensify their FCR:

*"When I hear someone talking about this disease (cancer), fear appears and persists"(P1). “When I hear about what happened to others (wardmate), I always feel like the same thing will happen to me" (P2).*

When survivors perceived a lack of understanding from the people around them, it could contribute to their FCR:

*"I feel that even the closest people in this world cannot empathize...When I just finished chemotherapy, I had a strong aversion to food and eating, but my wife could not understand. They said 'Why can other people eat during chemotherapy, and you can't even eat a bowl of porridge?" (P7).*

Family members concealing information, avoiding communication, or providing minimal support can significantly contribute to the FCR among the survivors:

*"My family still avoids me when they do things, and there are some emotions"(P14). "Everyone avoids me when eating now, afraid that I will infect them because I am a patient"(P12).*

When the survivors needed to rely on the people around them, it amplified their FCR:

*“I am afraid that I will not be able to get up in the future and will need to rely heavily on my husband"(P5).*

The alienation and cancer stigma experienced from the people around them were additional factors that contributed to the FCR among the survivors:

*“After I got sick, most of my friends did not interact with me much. Before, everyone came to visit when I was sick because it was a common disease that could be cured. After I got cancer, almost no one came, and I thought about progression and death"(P19). "There are many things I do not understand, and I do not dare to ask others or doctors because I am afraid of bothering them if I ask too much"(P14).*

#### 3.2.4 The stress arising from making future plans

When making plans for the future, the survivors often adopted a pessimistic attitude. Some expressed the belief that future arrangements hold little meaning, influenced by their FCR and uncertainty about their health:

*"I am particularly worried that my partner will not be able to take care of himself if he gets sick in the future"(P20). "I am afraid that I will not have a job in the future and will have to take medication for the rest of my life"(P9). “I don't think too much about the future. I just live foolishly. I don't know when cancer will progress"(P2).*

The majority of survivors mentioned that thoughts of economic pressure tended to trigger fear more readily. This was particularly true for caregivers who had made the decision to give up their jobs in order to provide care for the survivor:

*"I am particularly worried about the economy. Our house was demolished, and I'm afraid that treatment will be a bottomless pit"(P6)."We have medical insurance in our family, but we were hospitalized more this year. I also want doctors and nurses to try their best to help us save money"(P14). "Neither of us works now, and the economy is a bit behind. Because if one person works, many things cannot be done, so someone needs to stay home to take care of me"(P9).*

### 3.3 Coping strategies for the FCR: positive coping and negative coping coexist

#### 3.3.1 Avoidance of fear, autonomously

Some survivors believed that avoiding any contact with anything related to cancer is an effective way to prevent fear from arising:

*"I don't go online or watch anything related to cancer" (P1). "I don't watch TV shows or programs related to cancer patients" (P2).*

They also chose to minimize social activities and work commitments in order to avoid additional stress and alleviate their fear:

*"I have reduced my gatherings with friends and relatives to avoid stress" (P6). "When I feel stressed, I may become more afraid of progression, so I choose not to work" (P9).*

Some survivors voluntarily chose to reduce the frequency of medical examination to alleviate their anxiety:

*"I'm always worried when I have a check-up. I used to have X-rays taken every time I had a follow-up, but now I don't" (P6).*

#### 3.3.2 Self-endurance

When confronted with the FCR, the majority of the respondents chose to keep their concerns to themselves and silently endure the emotional burden. Some respondents expressed that their decision to remain silent was to avoid adding psychological burden to others:

*"Your illness is your own, you have to bear it yourself. So, no matter how afraid or uncomfortable you are, you have to bear it yourself. I don't tell others about it. I just endure it on my own” (P13). "Like this time, when the white blood cell test results were not good (choking). How should I say it? I'm very worried, but you can only keep it to yourself... and endure it (choking again)." (P9). "I usually don't talk to my partner about it. I feel like telling her would only increase her burden......I usually do things on my own and don't let my wife help me. I rarely talk to her about my illness, because women have weaker acceptance abilities” (P7).*

#### 3.3.3 Accepting reality

Most of the survivors have come to accept the reality of cancer and have learned to coexist with their fear:

*“The biggest feeling of illness is to leave it to fate......Since I'm seeking treatment now, I must try to accept it all...... "(P16).*

Some survivors have accepted the possibility of cancer progression, but have adopted negative coping methods:

*"There's nothing to say, it's best to live like a fool"(P1). “Now that I think about it, we all have to die sooner or later. There's no way to avoid it"(P20).*

When fear comes to their minds, the survivors often try to keep themselves busy as a means of distracting their attention and diverting their focus away from their fears:

*"I have children to take care of, and a lot of work, so I don't have time to worry” (P7)." I will try to keep myself busy, like doing housework or going for a walk" (P8).*

By engaging in activities they enjoyed and consciously adjusting their mindset, the survivors strived to alleviate their fear and regain a sense of control over their emotions:

*"As long as I do things I like, I won't think too much, or distract myself" (P14). "Many times when I'm in a bad mood or afraid, I will go play Card Landlord to relieve myself" (P8).*

#### 3.3.4 Healthy belief and lifestyle

Positive cognitive-behavioral coping strategies can effectively regulate negative emotions. The survivors actively took preventive measures to reduce the risk of cancer progression by cultivating healthy lifestyle habits:

*"Establishing my own circle. Everyone comes to me to mediate family conflicts. They all trust me and rely on me. I feel they all need me, so I am satisfied" (P19).*

The survivors paid particular attention to their diet due to gastrointestinal symptoms:

*"I don't eat pickled food anymore. I'm very careful about my diet, eat less, don't eat cold food, and even scald small oranges before eating them. Then I usually pay attention to eating high-protein foods, which can reduce the possibility of progression" (P3).*

#### 3.3.5 Seeking help and support from family members

Family members played an important role in assisting and supervising the survivors in developing and maintaining healthy lifestyle habits that helped reduce the risk of cancer progression, and caregivers also made changes to their own unhealthy habits in consideration of the survivors’ health:

*"My wife doesn't allow me to smoke or drink. I used to drink a lot of alcohol a day, but now I've quit and don't drink as much anymore" (P12).*

Due to the severe gastrointestinal symptoms in survivors of gastric cancer, family members provided meticulous care for them and paid extra attention to their diet:

*"Now I eat well every day. My spouse makes a lot of nutritious food for me to eat" (P4). "Family members make nutritious soup for me and give me vitamins to eat" (P6).*

The psychological comfort provided by family members was essential for alleviating the fear experienced by survivors. Some survivors shared their feelings with their partners to alleviate their anxiety:

*"I talk to my partner when there's a problem, and he comforts me a lot" (P6). "He often encourages me and says kind words to me. I am very grateful to my wife, who has worked hard all her life" (P14).*

Intergenerational communication and support serve as effective ways for them to overcome their fears:

*“My son is very capable, and he makes me proud. This is also the motivation for me to keep going in my treatment" (P16). "My family will try their best to support me financially, and it makes me feel at ease" (P8).*

#### 3.3.6 Emotional venting

Some survivors used venting as a way to alleviate their anxiety with their caregivers:

*"Sometimes when I'm scared, I get angry with the people around me, but I feel better after I vent" (P13).*

#### 3.3.7 Seeking professional and social support

When the survivors found themselves unable to confront their fears alone, seeking necessary social support became crucial to changing the situation. Oncologists and attending physicians were often regarded as the most reliable sources of disease-related information:

*"I went to Shanghai (a higher-level hospital) to see a specialist in the oncology department for an examination. I never let a general practitioner examine me, but go directly to see a cancer specialist. This can avoid a lot of anxiety. The accurate information provided by professionals can often give me peace of mind" (P1).*

At the same time, the survivors firmly believed that the care and emotional support provided by nurses were immensely helpful in alleviating their psychological fears:

*"The benefit is that we now have the help of a nurse, which is very helpful for many things, such as our life and psychological adjustment. She has introduced us to many new methods" (P7)*

Good peer support can also provide psychological relief for the survivors, and help establish meaningful friendships with other fellow survivors:

*“We were all diagnosed with stomach cancer, so we can rely on each other and have empathy for each other" (P1).*

## 4 Discussion

### 4.1 The cancer progression of survivors with gastric cancer manifests as various irrational fears and is influenced by multiple factors, such as family and environment

This study revealed that survivors of cancer experience complex fears related to disease progression. These fears manifest in various dimensions. In terms of the cognitive dimension, survivors exhibit uncontrollable and irrational thoughts. Emotionally, they experience intense and persistent fear and anxiety. On the physical dimension, they display a state of suspicion and vigilance, doubting treatment efficacy and fearing medical examination results. These findings are consistent with the research conducted by Mutsaers et al. (21).

The study also supports Ellis’ ABC theory of emotion, which suggests that various negative emotions arise from negative cognitions associated with stressful events (22). Therefore, survivors’ intense and persistent fear and anxiety, along with somatic symptoms like tension and insomnia, may be attributed to overly pessimistic and irrational beliefs about their disease prognosis. Furthermore, this study identified that survivors’ family members also experience worry and fear about the survivor’s physical condition. Conversely, survivors worry about how the disease progression affects their family’s normal life, aligning with the results of previous research findings (23). Patients are often hesitant to burden their families with increased psychological and financial strain. This fear is influenced by the value concept of “unwillingness to trouble others and prioritizing collective interests,” which is prevalent in traditional Chinese culture.

Sahin et al. (24) reported that approximately 50.8% of lymphoma patients and 57.6% of their caregivers experienced high levels of FCR (FCR). Although the cancer type differs, their findings suggest that many influencing factors—such as shorter time since diagnosis, higher anxiety, lower quality of life, and higher caregiver FCR—are common across different cancer populations. Similar associations were observed in our study on gastric cancer survival. However, the current research did not include the caregiver dimension of FCR. Sahin et al. also identified caregiver FCR as a significant predictor of patient FCR, underscoring the importance of incorporating family or caregiver perspectives into future survivorship research on gastric cancer in China. Furthermore, their recommendation to implement comprehensive nursing interventions and early screening mechanisms provides valuable implications for enhancing psychological support during the survivorship phase among Chinese gastric cancer patients.

Pang and Humphris (25), through a systematic review and meta-analysis, found that female cancer survivors tend to report slightly higher levels of FCR than males, a pattern consistently observed across various cancer types. This suggests that women may be more prone to experiencing recurrence-related anxiety. Although gender differences were not specifically analyzed in our study, the current sample included 15 female and five male participants. This gender imbalance could be informative, particularly given existing evidence indicating that gender differences may influence patterns of fear of recurrence or progression.

### 4.2 Provide reliable information around triggering factors to eliminate the survivor’s disease uncertainty

Most of the existing research on FCR has primarily focused on exploring its influencing factors, with fewer studies specifically examining the triggering factors of FCR (11). The results of this study indicate that survivors’ FCR can be triggered by various factors, including their perception of their own symptoms and physical changes, cancer-related treatments and examinations, interactions with healthcare providers, the influence of the surrounding environment, the lack of understanding from family and friends, and the pressure of planning for the future.

According to Mishel’s theory of disease uncertainty (26), uncertainty about information such as diagnosis, treatment, and prognosis can contribute to disease-related uncertainty, which can, in turn, trigger feelings of fear. If survivors do not have a correct understanding of cancer treatment, prognosis, or death, they may experience disease uncertainty when they notice signs of disease progression, as they lack reliable information about the disease. Additionally, when survivors are faced with future planning or discussions about cancer with those around them, they may enter an irrational cognitive state, becoming overly pessimistic about the future and experiencing negative emotions. This study also corroborated these findings, indicating that the triggering factors—such as symptoms and vague communication—are manifestations of ambiguity and lack of information, which constitute the core components of illness uncertainty.

The lack of empathy from healthcare providers, family, and friends can also contribute to survivors with cancer feeling misunderstood and lonely, which aligns with the findings of previous research conducted by Rosedale et al. (27) and Raque-Bogdan et al. (28). Some interviewees in this study also reported perceiving neglect from their family members and friends’ alienation. Such experiences not only hurt the emotional well-being of cancer survivors but also make them more sensitive and vigilant, lacking an outlet to express their feelings. These findings highlight the importance of addressing the triggering factors of FCR and the need for healthcare providers, family, and friends to provide empathy, support, and understanding to survivors with cancer. By addressing these factors, it is possible to alleviate survivors’ fear and promote their overall well-being.

### 4.3 Develop a comprehensive hospital-family-society support model to help survivors improve coping strategies, accept the disease, and take positive actions

The findings of this study suggest that silence and avoidance are commonly employed coping strategies among survivors of gastric cancer. However, it is important to note that avoidance coping may only provide temporary relief from stress, as previous research has demonstrated its ineffectiveness in alleviating disease uncertainty (29). In fact, relying on avoidance coping strategies can lead to the accumulation of stress, the emergence of new problems, and the development of more severe psychological symptoms such as fear, anxiety, or depression (30).

The inclination toward self-endurance may stem from a cultural influence, specifically the emphasis on endurance within traditional Chinese culture (31). In an effort to avoid burdening others with negative emotions, survivors may choose to suppress their own emotions and endure physical and mental pain in isolation (32). Similar observations have been made by Adams et al. (33), who reported instances of concealing illness information for protective reasons. However, excessive self-endurance is not conducive to the psychological well-being of cancer survivors.

For survivors who cope with fear alone through avoidance, healthcare providers should implement measures that address the triggers of fear. These measures may include providing accurate medical information, involving survivors in the decision-making process, and ensuring their right to make informed choices about their healthcare. Alongside emotional support, healthcare providers can actively engage in health education (34) and organize peer support activities (35) to help survivors build confidence in facing their illnesses. Additionally, acceptance and commitment therapy can be employed to enhance survivors’ psychological flexibility, reduce maladaptive coping styles, and prevent avoidance behaviors. It can also guide survivors in adjusting their mindset, encourage marital self-disclosure, and help them incorporate appropriate social activities into their schedules (taking into consideration not causing excessive fatigue) (36). When circumstances do not allow for in-person interactions, online social communication can serve as a supplement to interpersonal communication to address negative emotions (37, 38).

In traditional Chinese culture, which emphasizes a “family-oriented” approach, the family plays a crucial role in emotional connections, establishing family rules, facilitating family communication, and coping with external events. The care of cancer survivors has become a significant issue that requires the collective effort of the entire family. Family support, as an available and effective resource, has been proven to be an important resource in promoting psychological adjustment, alleviating symptom distress, and reducing FCR among survivors of cancer (39, 40).

To effectively support the families of survivors, healthcare professionals are recommended to provide comprehensive psychological care that addresses the needs of both the survivor and their relatives. This involves teaching family members how to provide survivors with cancer with adequate companionship and care to survivors with cancer, creating a warm and open family communication atmosphere, and encouraging survivors to express their inner thoughts and feelings. It is also important for survivors to cultivate and develop hobbies and interests, carefully plan their daily lives well, and try to maintain a regular and fulfilling routine while doing things that are within their capabilities and meaningful to them. Research by Parker et al. (41) demonstrated that doctors’ attentive listening and empathy were effective in alleviating negative emotions among survivors. The study also revealed that interventions based on Solution-Focused Therapy (SFT), the Benson Relaxation Technique, and Music Therapy (42, 43) have demonstrated effectiveness in alleviating stress, anxiety, depression, somatization, cancer-related fatigue, pain catastrophizing, and negative coping. However, research addressing FCR remains limited. Future studies should employ a series of methodological approaches to further verify the effectiveness of these interventions in reducing FCR.

Clinical healthcare professionals should guide survivors in adopting positive coping strategies, proactively understand and address their supportive care needs, and minimize disease uncertainty and FCR. This includes providing sufficient disease-related information, support, and professional personalized rehabilitation guidance. Furthermore, healthcare professionals should actively advocate for improvements in social systems and public policies that specifically target survivors of cancer. By implementing these recommendations, healthcare professionals can contribute to the wellbeing of survivors and their families, promote effective coping strategies, mitigate fears, and create a supportive environment that fosters resilience and overall psychological adjustment. Given that FCR or progression (FCR/FoP) is closely associated with psychological distress and quality of life, intervention strategies should not be confined to hospital-based settings but should also actively incorporate family and social support factors to more effectively alleviate patients’ FCR. Moreover, psychometric validation of FCR measurement tools across different cultural contexts remains essential (44–46). Future studies should consider conducting longitudinal validation of the *Fear of Progression Questionnaire–Short Form (FoP-Q-SF)* among Chinese patients with gastric cancer, while integrating caregiver or family perspectives and sociocultural variables to further refine and strengthen intervention models.

## 5 Strengths and limitations

The strengths of this qualitative study presented several strengths that contributed to the reliability and depth of the results. The rigorous scientific process and strict quality control measures ensure the credibility of the study. The interview guide was developed through expert consultation and pre-interviews, enhancing its validity and appropriateness. The determination of sample size based on data saturation strengthens the comprehensiveness of the findings. Conducting interviews prior to chemotherapy administration minimizes the potential influences of adverse chemotherapy drug reactions on participants’ experiences. The strict control of interview time was maintained, and prompt transcription of the interviews was recorded and transcribed within 24 h to enhance the accuracy of data collection.

The study’s focus on survivors diagnosed with gastric cancer and the in-depth exploration of their FCR provide valuable insights into this specific population. The investigation of triggers and the impact of survivors’ family members, friends, and the environment on the FCR adds depth to the understanding of survivors’ experiences. The analysis of positive and negative coping strategies employed by survivors contributes to the development of effective support models.

However, it is important to acknowledge some limitations. The study’s generalizability may be limited as participants were primarily recruited from a single hospital’s oncology department. Future research should aim to include a more ethnically diverse range of hospitals. It is important to account for potential variations in experiences and perceptions. The presence of selection bias should also be considered, as the study may have inadvertently excluded individuals with limited resources or alternative coping strategies. Longitudinal data collection could provide valuable insights into the evolving experiences and coping strategies of survivors of gastric cancer. Overall, the study’s strengths and limitations provide a foundation for future research to further explore and address the complex psychological experiences and needs of survivors of gastric cancer. This study only explored the experiences of FCR among gastric cancer patients within the context of Chinese culture. Therefore, the findings may not be generalizable to patients from other cultural or healthcare settings. In addition, as qualitative interviews were conducted by a single research team, potential interviewer bias cannot be fully excluded. Furthermore, the study did not include longitudinal follow-up; hence, changes in FCR over time could not be captured.

## Data Availability

The original contributions presented in the study are included in the article/supplementary material, further inquiries can be directed to the corresponding author/s.
